# Different effects of low muscle mass on the risk of non‐alcoholic fatty liver disease and hepatic fibrosis in a prospective cohort

**DOI:** 10.1002/jcsm.13125

**Published:** 2022-11-20

**Authors:** Hun Jee Choe, Hyunsuk Lee, DongHo Lee, Soo‐Heon Kwak, Bo Kyung Koo

**Affiliations:** ^1^ Department of Internal Medicine Seoul National University College of Medicine Seoul South Korea; ^2^ Department of Internal Medicine, Seoul National University Hospital Seoul South Korea; ^3^ Department of Internal Medicine Seoul Metropolitan Government Boramae Medical Center Seoul South Korea

**Keywords:** Non‐alcoholic fatty liver disease, Liver fibrosis, Sarcopenia, *PNPLA3*, *TM6SF2*

## Abstract

**Background:**

Non‐alcoholic fatty liver disease (NAFLD) and sarcopenia share insulin resistance as a common pathophysiology and have overlapping clinical manifestation of metabolic derangement; hence, it is difficult to differentiate the independent effect of sarcopenia on the development of NAFLD from concomitant metabolic disorders. Using a community‐based prospective cohort study, the contributions of low muscle mass and genetic risk factors to the development of NAFLD and NAFLD‐related hepatic fibrosis were investigated in the Korean population.

**Methods:**

This prospective community‐based cohort study included 40–70‐year‐old adults, followed up biennially from 2001–2002 to 2017–2018. NAFLD was defined as a hepatic steatosis index of ≥36, and hepatic fibrosis was defined based on the fibrosis‐4 index. Sex‐specific quartiles of body mass index (BMI)‐adjusted muscle mass were calculated (muscle mass/BMI), and low muscle mass was defined as the lowest quartile (Q1). Cox proportional hazard models for incident NAFLD or hepatic fibrosis incorporating age, sex, BMI of ≥25 kg/m^2^, metabolic syndrome and *PNPLA3* and *TM6SF2* risk alleles were used to assess the independent determinants for incident NAFLD and hepatic fibrosis among individuals with NAFLD at baseline.

**Results:**

Among the 4038 participants without NAFLD at baseline (mean age, 51.5 ± 8.8 years), 920 (22.8%) developed NAFLD during the 12‐year follow‐up period. As muscle mass decreased, the risk of NAFLD increased even after adjustment for age, sex, obesity, metabolic syndrome and *PNPLA3* and *TM6SF2* risk alleles [hazard ratio (HR) per quartile, 1.18, 95% confidence interval (CI), 1.11–1.27, *P* < 0.001]. *TM6SF2* also affected the risk of NAFLD development [HR 1.19, (95% CI, 1.00–1.40), *P* = 0.044].

Of the 1176 patients with NAFLD but without hepatic fibrosis at baseline, the incident of hepatic fibrosis was found in 51.8%, 44.7%, 42.6% and 41.0% in Q1, Q2, Q3 and Q4 of BMI‐adjusted muscle mass, respectively, during the follow‐up period (*P* for trend = 0.006). However, this trend lost its statistical significance when adjusted for confounders. The *PNPLA3* risk variant, but not the *TM6SF2* genotype, was an independent risk factor for developing hepatic fibrosis among patients with NAFLD (HR 1.17, 95% CI 1.04–1.32, *P* = 0.010).

**Conclusions:**

Both lower muscle mass index and genetic risk variants are important contributors to the development of NAFLD. In patients already diagnosed with NAFLD, however, *PNPLA3* confers a greater risk for hepatic fibrosis progression than lower muscle mass.

## Introduction

The global prevalence of non‐alcoholic fatty liver disease (NAFLD) is incessantly rising and constitutes more than 25% of the population in today's obesogenic society.^1^ Currently, NAFLD is the most common cause of chronic liver disease, increasing the clinical burden of not only liver‐related complications but also of extrahepatic manifestations including cardiovascular diseases and mortality.^2^

To date, the stage of hepatic fibrosis is perceived as the most critical determinant of mortality in patients with NAFLD.^3^, ^4^ Contemporaneous manifestations of sarcopenia and NAFLD‐related hepatic fibrosis have been reported in several cross‐sectional studies,^5^, ^6^, ^7^, ^8^, ^9^, ^10^ as well as the association between sarcopenia and NAFLD.^11^, ^12^ As sarcopenia and NAFLD‐related hepatic fibrosis both naturally increase with age and share common chronic catabolic conditions, it is difficult to determine the causality between sarcopenia and NAFLD‐related hepatic fibrosis. To the best of our knowledge, studies that explored the independent contribution of low muscle mass to the development of NAFLD‐related hepatic fibrosis in prospective cohorts are scarce.

NAFLD is a multisystem disease with a heterogeneous nature; both genetic and environmental factors influence its development and progression. Aside from insulin resistance and obesity, which are the leading pathogeneses of NAFLD,^13^ a strong association between liver fat and genetic variants in patatin‐like phospholipase domain‐containing protein 3 (*PNPLA3*) rs738409 and transmembrane 6 superfamily 2 human gene (*TM6SF2*) rs58542926 has been discovered through genome‐wide association studies.^14^, ^15^

We aimed to investigate the independent effects of low muscle mass on the incidence of NAFLD and NAFLD‐related hepatic fibrosis, adjusting for metabolic abnormalities and genetic risk factors in a prospective community‐based cohort study.

## Methods

### The Korean Genome and Epidemiology Study (KoGES) Ansung–Ansan cohort

The Korean Genome and Epidemiology Study (KoGES) Ansung–Ansan prospective cohort study was designed to investigate the genetic and environmental factors in chronic diseases in Koreans.^16^ Baseline recruitment for the Ansung–Ansan study was conducted in 2001–2002, and participants were followed biennially until 2017–2018. Participants were surveyed by trained interviewers at baseline and follow‐up; anthropometric measurements, laboratory tests and bioimpedance analysis to monitor body composition were repeated every 2 years.

The participants were 40–70 years old at baseline and were either from the Ansung (*n* = 4205) or Ansan (n = 4635) communities. We excluded individuals with a history of atherosclerotic cardiovascular disease, heart failure, cancer or thyroid disease, those with thyroid stimulating hormone levels lower than 0.4 IU/mL or higher than 5.0 IU/mL and individuals who reported steroid use or excessive alcohol intake (male >30 g/day, female >20 g/day), as this could adversely affect liver function independently of excess liver fat (*n* = 1602). After further exclusion of participants without bioelectrical impedance analysis (*n* = 1494) and genotype information (*n* = 326), a total of 5480 participants were finally included in the analysis.

This study was conducted in accordance with the Declaration of Helsinki, and the study protocol was approved by the ethics committee of the Korean Center for Disease Control and the Institutional Review Board of Ajou University School of Medicine (IRB number: AJIRB‐BMR‐SMP‐17‐477). All participants provided written informed consent.

### Measurement of metabolic parameters

Height, weight and waist circumference were measured on every visit by trained examiners. Blood samples were obtained after at least 12 h of overnight fasting and analysed using standard methods.

Diabetes mellitus was defined as a glycated haemoglobin level ≥6.5%, fasting plasma glucose level ≥126 mg/dL or plasma glucose concentration ≥200 mg/dL during a 75 g oral glucose tolerance test or as taking anti‐diabetic medications. Hypertension was defined as a systolic blood pressure ≥140 mmHg, diastolic blood pressure ≥90 mmHg or taking anti‐hypertensive medications. Obesity was defined as body mass index (BMI) of ≥25 kg/m^2^.^17^ Metabolic syndrome was considered to be present if three or more of the five criteria were met according to the National Cholesterol Education Program Adult Treatment Panel III (NCEP ATP III) criteria^18^: waist circumference over ≥90 cm (male) or 85 cm (female), blood pressure ≥130/85 mmHg, fasting triglyceride level ≥150 mg/dL, fasting high‐density lipoprotein (HDL) cholesterol level <40 mg/dL (male) or 50 mg/dL (female) and fasting blood glucose ≥100 mg/dL.

### Assessment of muscle mass

Body muscle mass was also measured using the bioimpedance method (Zeus 9.9; CELLA Healthcare, Seoul, South Korea) at every visit. Muscle mass, which is proportional to height (cm^2^) divided by resistance (Ω), was calculated within the machine with a specific regression equation using the following variables: height, bioimpedance resistance, body weight, age and sex.^19^ The participants were divided into sex‐specific quartiles according to BMI‐adjusted muscle mass (muscle mass/BMI). Low muscle mass was defined as the lowest quartile (Q1) of the BMI‐adjusted muscle mass groups.

### Definition of NAFLD and hepatic fibrosis

NAFLD was diagnosed based on hepatic steatosis index (HSI), a well‐validated biomarker to diagnose hepatic steatosis.^20^ HSI was calculated as 8 × aspartate aminotransferase (AST)/alanine aminotransferase (ALT) + BMI (+2, if female; +2, if diabetic),^20^ and those with HSI > 36 were defined to have NAFLD.

Hepatic fibrosis was assessed using the fibrosis‐4 index (FIB‐4), calculated as age (year) × [AST/platelet count (10^9^/L)] × AST^(1/2)^.^21^ FIB‐4 index ≥1.3 in underlying NAFLD patients was defined as having hepatic fibrosis.

AST to platelet ratio index (APRI), another non‐invasive diagnostic tool for assessing liver fibrosis, was used as an adjunct calculated as follows: AST/AST upper limit of normal/platelet count (10^9^/L) × 100, with AST upper limit of normal set as 40 IU/L and APRI ≥0.5 as a cut‐off for defining hepatic fibrosis.

### Genotyping

Genomic DNA was extracted from peripheral leukocytes and genotyped using the Affymetrix Genome‐Wide Human SNP Array 5.0.^22^ Imputation was performed using TOPMed Imputation Server (https://imputation.biodatacatalyst.nhlbi.nih.gov/).^23^

### Statistical analyses

Data are expressed as the mean ± standard deviation for continuous variables that follow normal distribution, median (interquartile range [IQR]) for data not normally distributed and numbers and percentages for categorical variables. Differences among groups were evaluated using Student's *t*‐test, analysis of variance or Mann–Whitney non‐parametric *U* test for continuous variables and the chi‐squared test for categorical variables. For data *P* for trends was calculated using polynomial regression for continuous variables and the Cochran–Armitage test for categorical variables for comparing variables in BMI‐adjusted muscle mass quartiles.

The risk of developing NAFLD or hepatic fibrosis was assessed using Cox proportional hazard models to estimate the hazard ratios (HR) and 95% confidence intervals (CI). All analyses were performed using IBM SPSS Statistics version 27.0 (IBM Corp., Armonk, NY, USA) and R version 4.1.2 (The R Foundation for Statistical Computing, Vienna, Austria, http://www.R‐project.org). A two‐sided *P* value of <0.05 was considered statistically significant.

## Results

### Baseline characteristics of the study participants

**Figure 1:**
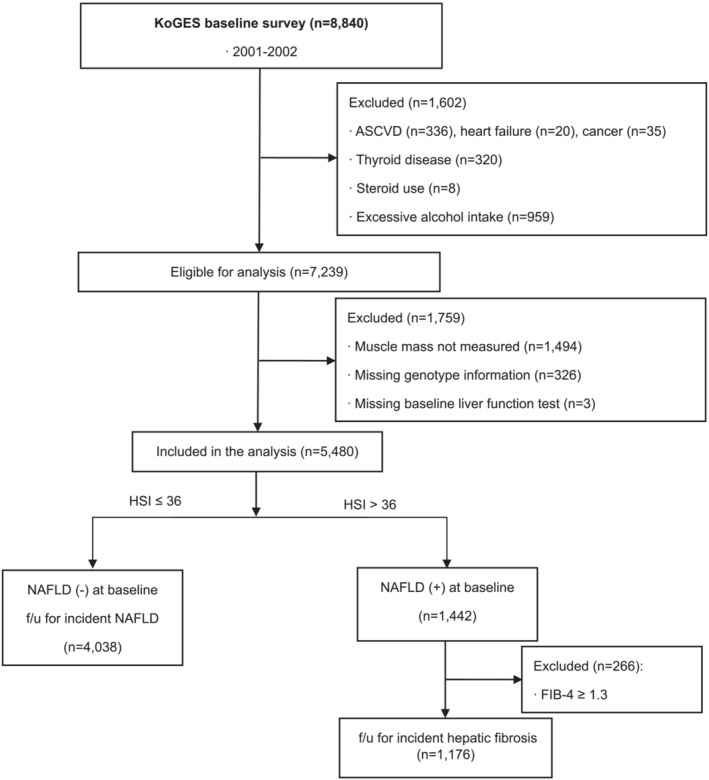
Consort diagram of participants enrolled in the Korean Genome and Epidemiology Study (KoGES). ASCVD, atherosclerotic cardiovascular disease; f/u, follow‐up; FIB‐4, fibrosis‐4 index score; HSI, hepatic steatosis index; NAFLD, non‐alcoholic fatty liver disease; excessive alcohol intake was defined as alcohol consumption of >30 g/day for male and >20 g/day for female.

We analysed 5480 participants with muscle mass data and genotype information at baseline (*Figure* 1). The mean age of the total study participants was 51.5 ± 8.8 years, and 3078 (56.3%) were female (*Tables* 1 and S1). Participants with NAFLD at baseline consisted of more female (59.5% vs 55.1%) and had higher rates of hypertension, diabetes mellitus, metabolic syndrome and obesity (*Table* 1). However, the BMI‐adjusted muscle mass was significantly lower in participants who had NAFLD at baseline (1.64 ± 0.32 vs 1.80 ± 0.32, *P* < 0.001).

**Table 1:** Characteristics of participants according to baseline NAFLD

	Total	NAFLD (−)	NAFLD (+)	*P* value
	*N* = 5480	N = 4038	*N* = 1442	
Age	51.5 ± 8.8	51.5 ± 8.9	51.7 ± 8.5	0.417
Female (*n*, %)	3087 (56.3)	2223 (55.1)	864 (59.9)	0.001
Smoking (pack‐year)	0.0 (10.8)	0.0 (11.5)	0.0 (10.0)	0.018
Alcohol (g/day)	0.0 (3.7)	0.0 (4.3)	0.0 (2.7)	<0.001
Alcohol consumption duration (year)				0.601
0–5	306 (11.8)	234 (11.9)	72 (11.6)	
6–10	283 (10.9)	220 (11.2)	63 (10.1)	
11–20	476 (18.4)	351 (17.8)	125 (20.1)	
≥21	1525 (58.9)	1162 (59.1)	363 (58.3)	
Hypertension	1343 (24.5)	843 (20.9)	500 (34.7)	<0.001
Diabetes mellitus	704 (12.8)	295 (7.3)	409 (28.4)	<0.001
Metabolic syndrome	2068 (37.7)	1063 (26.4)	1005 (69.7)	<0.001
Obesity (BMI ≥ 25 kg/m^2^)	2346 (42.8)	1050 (26.0)	1296 (89.9)	<0.001
Waist circumference (cm)	81.9 ± 8.8	79.2 ± 7.5	89.5 ± 7.4	<0.001
BMI (kg/m^2^)	24.6 ± 3.1	23.4 ± 2.3	27.9 ± 2.5	<0.001
Muscle mass/BMI	1.76 ± 0.33	1.80 ± 0.32	1.64 ± 0.32	<0.001
HbA1c (%)	5.8 ± 0.9	5.6 ± 0.7	6.2 ± 1.2	<0.001
Fasting glucose (mg/dL)	87.1 ± 21.6	84.7 ± 17.9	94.2 ± 28.6	<0.001
Platelet (×10^3^/μL)	267 ± 65	263.5 ± 63.8	277.6 ± 66	<0.001
Albumin (g/dL)	4.26 ± 0.33	4.26 ± 0.33	4.26 ± 0.33	0.658
Total bilirubin (mg/dL)	0.62 ± 0.32	0.63 ± 0.33	0.59 ± 0.29	<0.001
AST (IU/L)	26 (9)	25 (8)	28 (10)	<0.001
ALT (IU/L)	22 (14)	20 (10)	32 (22)	<0.001
GGT (IU/L)	18 (19)	15 (16)	27 (28)	<0.001
Total cholesterol (mg/dL)	192 ± 36	188 ± 35	203 ± 38	<0.001
Triglyceride (mg/dL)	133 (88)	123 (76)	169 (115)	<0.001
HDL‐C (mg/dL)	44 ± 10	45 ± 10	41 ± 9	<0.001
LDL‐C (mg/dL)	116 ± 33	114 ± 32	122 ± 35	<0.001
BUN (mg/dL)	14.3 ± 3.8	14.2 ± 3.7	14.6 ± 4.1	0.001
Creatinine (mg/dL)	0.84 ± 0.20	0.84 ± 0.2	0.85 ± 0.2	0.106
TSH (μIU/mL)	1.70 ± 0.91	1.74 ± 0.91	1.57 ± 0.92	0.206
Free T4 (ng/dL)	1.15 ± 0.17	1.14 ± 0.17	1.16 ± 0.19	0.504
CRP (mg/dL)	0.14 (0.18)	0.12 (0.16)	0.19 (0.22)	<0.001

Data are shown as mean ± standard deviation, median (interquartile range), or number (percentage).

ALT, alanine aminotransferase; AST, aspartate aminotransferase; BMI, body mass index; BUN, blood urea nitrogen; CRP, C‐reactive protein; GGT, γ‐glutamyl transferase; HDL‐C, high‐density lipoprotein‐cholesterol; LDL‐C, low‐density lipoprotein‐cholesterol; TSH, thyroid‐stimulating hormone.

Considering sex differences in muscle mass, we assessed the amount of muscle mass based on sex‐specific quartiles of BMI‐adjusted muscle mass. Lower muscle was associated with older age and higher prevalence of hypertension, diabetes, metabolic syndrome and obesity (*Table* S1).

### Association of low muscle mass and incident NAFLD

During the median follow‐up period of 12 years (interquartile range 4–14 years), 928 participants (22.8%) developed NAFLD out of those who did not have NAFLD at baseline (*n* = 4038). Individuals who developed NAFLD during the follow‐up were younger, but with higher rates of hypertension, diabetes mellitus, metabolic syndrome and obesity (*Table* 2).

**Table 2:** Baseline characteristics of participants according to the development of NAFLD

	Baseline		*P* value	Follow up		*P* value
	NAFLD (−)	NAFLD (+)		NAFLD (−)	NAFLD (+)	
	*N* = 3118	*N* = 920		*N* = 1846	*N* = 765	
Age	51.9 ± 9.0	50.0 ± 8.1	<0.001	65.0 ± 8.5	63.3 ± 7.9	<0.001
Female (*n*, %)	1657 (53.1)	566 (61.5)	<0.001	977 (52.8)	566 (61.5)	<0.001
Smoking (pack‐year)	0.0 (4.5)	0.0 (3.7)	<0.001	0.0 (10.5)	0.0 (7.0)	0.011
Alcohol (g/day)	0.0 (12.5)	0.0 (7.5)	0.812	0.0 (4.4)	0.0 (4.1)	0.800
Alcohol consumption duration (year)			0.099			0.016
0–5	170 (11.1)	64 (14.5)		31 (3.8)	17 (5.6)	
6–10	165 (10.8)	55 (12.5)		35 (4.3)	26 (8.6)	
11–20	283 (18.5)	68 (15.5)		79 (9.8)	32 (10.6)	
≥21	909 (59.5)	253 (57.5)		664 (82.1)	228 (75.2)	
Hypertension	619 (19.9)	224 (24.3)	0.003	614 (33.2)	351 (45.9)	<0.001
Diabetes mellitus	214 (6.9)	81 (8.8)	0.047	186 (10.1)	225 (29.4)	<0.001
Metabolic syndrome	723 (23.2)	340 (37.0)	<0.001	544 (29.6)	447 (58.4)	<0.001
Obesity (BMI ≥ 25 kg/m^2^)	576 (18.5)	474 (51.6)	<0.001	288 (15.6)	453 (59.4)	<0.001
Waist circumference (cm)	78.2 ± 7.5	82.3 ± 6.8	<0.001	81.8 ± 8.0	87.8 ± 7.7	<0.001
BMI (kg/m^2^)	23.0 ± 2.2	25.0 ± 1.8	<0.001	22.7 ± 2.3	25.5 ± 2.1	<0.001
Muscle mass/BMI	1.83 ± 0.32	1.72 ± 0.31	<0.001	1.76 ± 0.32	1.61 ± 0.31	<0.001
HbA1c (%)	5.6 ± 0.7	5.8 ± 0.9	<0.001	5.7 ± 0.6	6.0 ± 0.8	<0.001
Fasting glucose (mg/dL)	84.0 ± 16.1	86.8 ± 22.9	0.001	93.3 ± 15.9	101.1 ± 23.0	<0.001
Platelet (×10^3^/μL)	261.2 ± 63.8	271.0 ± 63.3	<0.001	241.1 ± 57.7	252.4 ± 58.3	<0.001
Albumin (g/dL)	4.25 ± 0.33	4.27 ± 0.32	0.102	NA	NA	NA
Total bilirubin (mg/dL)	0.63 ± 0.34	0.61 ± 0.30	0.093	NA	NA	NA
AST (IU/L)	26 (8)	25 (7)	0.066	23 (6)	24 (7)	0.114
ALT (IU/L)	20 (10)	21 (11)	0.009	18 (7)	23 (13)	<0.001
GGT (IU/L)	15 (11)	17 (17)	0.915	NA	NA	NA
Total cholesterol (mg/dL)	187 ± 35	192 ± 35	<0.001	188 ± 34	189 ± 37	0.662
Triglyceride (mg/dL)	119 (71)	136 (82)	<0.001	101 (63)	125 (85)	<0.001
HDL‐C (mg/dL)	46 ± 10	44 ± 9	<0.001	48 ± 13	44 ± 11	<0.001
LDL‐C (mg/dL)	113 ± 32	117 ± 33	0.001	116 ± 30	116 ± 33	0.992
BUN (mg/dL)	14.3 ± 3.8	14.1 ± 3.4	0.122	16.3 ± 4.8	16.3 ± 5.1	0.980
Creatinine (mg/dL)	0.84 ± 0.19	0.84 ± 0.23	0.732	0.98 ± 0.41	1.00 ± 0.48	0.439
TSH (μIU/mL)	1.79 ± 0.95	1.58 ± 0.78	0.201	1.94 ± 2.79	1.79 ± 1.26	0.175
Free T4 (ng/dL)	1.14 ± 0.17	1.15 ± 0.17	0.834	1.17 ± 0.18	1.19 ± 0.19	0.103
CRP (mg/dL)	0.12 (0.16)	0.13 (0.16)	0.275	0.56 (0.83)	0.73 (1.00)	<0.001
*PNPLA3* rs738409			0.838			0.655
CC	1056 (33.9)	316 (34.3)		604 (32.7)	264 (34.5)	
CG	1548 (49.6)	447 (48.6)		933 (50.5)	377 (49.3)	
GG	514 (16.5)	157 (17.1)		312 (16.9)	124 (16.2)	
*TM6SF2* rs58542926			0.145			0.171
CC	2609 (83.7)	751 (81.6)		1549 (83.8)	624 (81.6)	
CC + TT	509 (16.3)	169 (18.4)		300 (16.2)	141 (18.4)	

Data are shown as mean ± standard deviation, median (interquartile range), or number (percentage).

ALT, alanine aminotransferase; AST, aspartate aminotransferase; BMI, body mass index; BUN, blood urea nitrogen; CRP, C‐reactive protein; GGT, γ‐glutamyl transferase; HDL‐C, high‐density lipoprotein‐cholesterol; LDL‐C, low‐density lipoprotein‐cholesterol; NAFLD, non‐alcoholic fatty liver disease; TSH, thyroid‐stimulating hormone.

**Figure 2:**
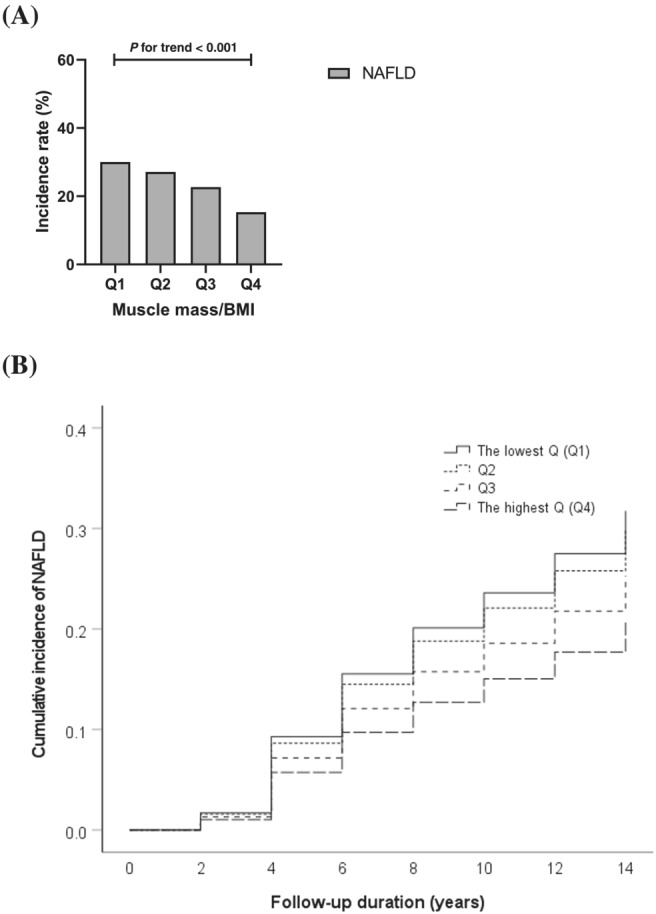
Incident rates of NAFLD according to BMI‐adjusted muscle mass quartiles. (A) Incident rate (%) of NAFLD according to BMI‐adjusted muscle mass quartile in bar graph. (B) Cumulative incidence of NAFLD during follow‐up in the fully adjusted model. Solid, dotted, dashed and long dashed line indicate the lowest quartile (Q1), Q2, Q3 and Q4, respectively. BMI, body mass index; NAFLD, non‐alcoholic fatty liver disease.

As BMI‐adjusted muscle mass increased, the incidence rate of NAFLD decreased: 30.0%, 27.1%, 22.7% and 15.3% in each quartile (*P* for trend <0.001; *Figure* 2A). Per 1 quartile decrease in BMI‐adjusted muscle mass, the risk of NAFLD significantly increased by 31% (HR, 1.31; 95% CI, 1.24–1.39; *Table* 3); the statistical significance of this was maintained after adjustment for age, sex, metabolic syndrome and obesity (Model 2 in *Table* 3). Even after additional adjustment for *PNPLA3* rs738409 and *TM6SF2* rs58542926 genotypes, low muscle mass significantly increased the risk of NAFLD (HR, 1.18; 95% CI, 1.11–1.27; Model 3 in *Table* 3). In the final model, the lowest quartile (Q1) had a 65% higher risk of NAFLD than the highest quartile (Q4) (HR, 1.65; 95% CI, 1.33–2.05; *Table* 3).

**Table 3:** Risk of NAFLD according to muscle mass/BMI quartile

	HR	95% CI	*P* value
Unadjusted			
Q1	2.30	1.89–2.79	<0.001
Q2	1.96	1.63–2.36	<0.001
Q3	1.53	1.27–1.85	<0.001
Q4	1 (reference)		
Per 1Q	1.31	1.24–1.39	<0.001
Model 1			
Q1	3.04	2.48–3.73	<0.001
Q2	2.29	1.89–2.76	<0.001
Q3	1.66	1.37–2.01	<0.001
Q4	1 (reference)		
Per 1Q	1.44	1.35–1.53	<0.001
Model 2			
Q1	1.66	1.34–2.07	<0.001
Q2	1.53	1.26–1.87	<0.001
Q3	1.26	1.04–1.53	0.019
Q4	1 (reference)		
Per 1Q	1.19	1.11–1.27	<0.001
Model 3			
Q1	1.65	1.33–2.05	<0.001
Q2	1.53	1.26–1.87	<0.001
Q3	1.26	1.04–1.53	0.020
Q4	1 (reference)		
Per 1Q	1.18	1.11–1.27	<0.001

Model 1: adjusted for age and sex. Model 2: adjusted for age, sex, metabolic syndrome and obesity. Model 3: adjusted for age, sex, metabolic syndrome, obesity and *PNPLA3*and *TM6SF2* genotypes.

CI, confidence interval; HR, hazard ratio; Q, quartile.

Among genotypes, only the dominant model of *TM6SF2* rs58542926 showed a modest association with the incident NAFLD in the final model (*Table* S2).

### Contributing factors in the progression of hepatic fibrosis

At baseline, 1442 (26.3%) participants had NAFLD (*Figure* 1). Of the 1176 participants with NAFLD but not liver cirrhosis, 552 (46.9%) eventually developed hepatic fibrosis using the FIB‐4 cut‐off of 1.3 (*Table* S3). Progression to hepatic fibrosis was associated with older age, but there was no difference between the sexes, and the prevalence of diabetes mellitus, metabolic syndrome and obesity at baseline did not differ between the participants who did or did not develop hepatic fibrosis (*Table* S3).

Incident of hepatic fibrosis was found in 51.8%, 44.7%, 42.6% and 41.0% in Q1, Q2, Q3 and Q4 of BMI‐adjusted muscle mass, respectively, during the follow‐up period (*P* for trend = 0.006), and the prevalence in Q1 was significantly higher than that in the other quartiles (51.8 vs 43.4%, *P* = 0.004).

**Figure 3:**
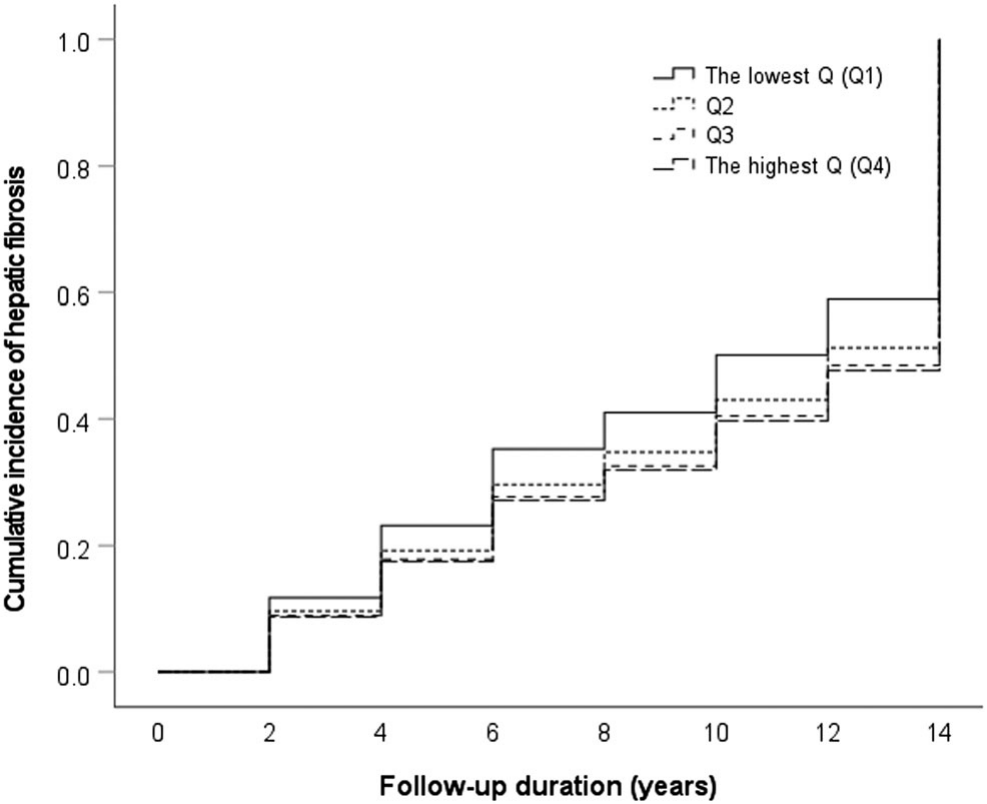
Development of NAFLD‐related hepatic fibrosis according to muscle mass quartiles. Cumulative incidence of NAFLD‐related hepatic fibrosis during follow‐up in the unadjusted model. Solid, dotted, dashed and long dashed line indicate the lowest quartile (Q1), Q2, Q3 and Q4, respectively. BMI, body mass index.

Cox proportional hazard models confirmed a negative relationship between the risk of incident hepatic fibrosis and muscle mass (HR per 1Q = 0.880, 95% CI 0.81–0.96, *P* = 0.004, *Figure* 3). The lowest BMI‐adjusted muscle mass or Q1 was significantly associated with higher risk for hepatic fibrosis development in the crude analysis (Q1 vs Q2–4, HR 1.29, 95% CI 1.09–1.53, *P* = 0.003; *Table* 4). For multivariable models, we controlled for age and sex in Model 1; age, sex, the presence of metabolic syndrome and obesity in Model 2; and age, sex, presence of metabolic syndrome, obesity and risk alleles of *PNPLA3* and *TM6SF2* in Model 3. Independent determinants for both the incident of NAFLD and hepatic fibrosis in participants with NAFLD at baseline were assessed using Model 3. Additionally, sensitivity analyses were performed by assessing the associations of Q1 in the development of hepatic fibrosis, in subgroups defined by the participants' age (<50 vs ≥50 years), sex (male vs female), the presence of metabolic syndrome, obesity (BMI < 25 vs ≥ 25 kg/m^2^) and the risk alleles of *PNPLA3* and *TM6SF2* using dominant models. The rate of progression to hepatic fibrosis was higher in Q1 compared with Q2–Q4 regardless of clinical factors in the subgroup analyses (*Figure* S1). However, this association was completely abolished after adjusting for age and sex (HR 1.00, 95% CI 0.84–1.20, *P* = 0.961, Model 1 in *Table* 4). In the fully adjusted model, the risk allele in *PNPLA3* rs738409 and older age and male sex were identified to be contributing factors for developing hepatic fibrosis (HR, 1.17; 95% CI, 1.04–1.32; HR, 1.07; 95% CI, 1.06–1.08; and HR, 1.36; 95% CI, 1.14–1.63, respectively; Model 3 in Table 4). Same trend was observed in participants' age < 65 years at baseline (*Table* S4). The effect of BMI‐adjusted muscle mass was also abrogated when hepatic fibrosis was evaluated using APRI (*Table* S5).

**Table 4:** The risk of progression to hepatic fibrosis according to muscle mass index

		HR	95% CI	*P* value
Unadjusted	Low muscle mass (Q1)	1.29	1.09–1.53	0.003
Model 1	Low muscle mass (Q1)	1.00	0.84–1.20	0.961
	Age	1.07	1.05–1.08	<0.001
	Sex	1.33	1.12–1.59	0.001
Model 2	Low muscle mass (Q1)	1.01	0.84–1.20	0.934
	Age	1.07	1.06–1.08	<0.001
	Sex	1.35	1.13–1.61	0.001
	Metabolic syndrome	0.82	0.68–0.98	0.032
	Obesity	1.01	0.76–1.34	0.944
Model 3	Low muscle mass (Q1)	1.02	0.85–1.22	0.843
	Age	1.07	1.06–1.08	<0.001
	Sex	1.36	1.14–1.63	0.001
	Metabolic syndrome	0.83	0.69–1.00	0.050
	Obesity	1.01	0.76–1.33	0.959
	*PNPLA3*	1.17	1.04–1.32	0.010
	*TM6SF2*	1.22	0.97–1.54	0.084

Model 1: adjusted for age and sex. Model 2: adjusted for age, sex, metabolic syndrome and obesity. Model 3: adjusted for age, sex, metabolic syndrome, obesity and *PNPLA3* and *TM6SF2* genotypes.

CI, confidence interval; HR, hazard ratio; Q, quartile.

## Discussion

NAFLD and NAFLD‐related hepatic fibrosis arise from a complex interplay of environmental and genetic factors. Here, we demonstrated that low muscle mass independently contributed to the development of NAFLD, in conjunction with risk alleles of *TM6SF2*. Association of low muscle mass with NAFLD‐related hepatic fibrosis was ameliorated, however, after adjusting for potential confounding factors. Risk alleles in *PNPLA3* additively conferred risk of hepatic fibrosis in participants with NAFLD at baseline, regardless of muscle mass or other metabolic risk factors.

Accumulating evidence suggests sarcopenia as an important risk factor for NAFLD.^24^, ^25^ Sarcopenia and NAFLD share common pathophysiological mechanisms, for example, age, insulin resistance, obesity, low‐grade inflammation and sedentary lifestyle.^26^ In this longitudinal study, we were able to demonstrate that low BMI‐adjusted muscle mass is not only more prevalent in participants with underlying NAFLD but also an independent risk factor for the subsequent development of NAFLD in Korean adults. Each decrease in the BMI‐adjusted muscle mass quartile conferred a higher risk of incident NAFLD after controlling for other metabolic derangement. The importance of preserving adequate skeletal muscle mass cannot be emphasized enough, as sarcopenia and NAFLD may additively increase mortality.^27^

NAFLD is an umbrella term encompassing a broad spectrum from benign steatosis to non‐alcoholic steatohepatitis with various degrees of hepatocyte injury and fibrosis.^28^ In line with the perspective that NAFLD without fibrosis is innocuous per se, we endeavoured to ascertain whether lower muscle mass also contributed to the development of hepatic fibrosis in patients diagnosed with NAFLD but did not have liver cirrhosis at baseline. Despite the apparent relationship between the lowest BMI‐adjusted muscle mass quartile (Q1) and incident hepatic fibrosis, this association was outweighed by other parameters when adjusted for baseline demographic factors. Taking into account the much higher prevalence of sarcopenia in patients with liver cirrhosis than in the general population, it may be more appropriate to consider sarcopenia a corollary of liver cirrhosis. The presence of liver cirrhosis contributes to the development of sarcopenia via various factors: portal hypertension complications, elevated hepatic insulin resistance and pro‐inflammatory cytokines.^29^

Individual susceptibility to NAFLD and NAFLD‐related hepatic fibrosis is highly variable owing to the genetic component. Notably, genetic polymorphism of *TM6SF2* in the dominant model was independently linked to the risk of developing NAFLD, and *PNPLA3* genetic variants additively increased vulnerability to NAFLD‐related hepatic fibrosis. Both risk variants in *PNPLA3* and *TM6SF2* are infamous for increased hepatic fat content, by regulating lipid droplet triglyceride mobilization and modulating the secretion of very low‐density lipoprotein, respectively.^30^ Later, based on a Mendelian randomized approach, which proposed that *PNPLA3* I148M does not causally conduce to ischemic heart disease, genetic variability in *PNPLA3* appeared to be cleared from the notorious frame of exacerbating chronic liver diseases.^31^ Because studies exhibit robust association between PNPLA3 and the severity of NAFLD, the debate is ongoing, and further studies are warranted to better elucidate the true role of *PNPLA3* I148M in sequelae that may arise in conjunction to NAFLD.^32^

In the current study, we have determined that participants with *PNPLA3* risk variants are more susceptible to developing NAFLD‐related hepatic fibrosis, after adjusting for confounders including muscle mass. This is in accordance with a recent study that demonstrated that silencing of *PNPLA3* could effectively attenuate hepatic steatosis as well as reduce liver fibrosis via lowering Timp2 expression levels.^33^ A clinical implication from this finding is that genetic susceptibility may override modifiable environmental factors in patients diagnosed with NAFLD and patients with risk alleles in *PNPLA3* may benefit from additional intensive surveillance to prevent hepatic fibrosis progression.

This study has a few limitations. First, diagnosis of NAFLD and hepatic fibrosis were based on non‐invasive screening tools. Liver biopsy is the gold standard for definitive diagnosis, and non‐invasive screening tools, albeit well validated, were not developed with the intention to substitute for liver biopsy. The variables used to define NAFLD and hepatic fibrosis might have overvalued the involved risk factors. However, it is extremely challenging to perform biannual liver biopsies or imaging studies with a large‐scaled community‐based cohorts. Similarly, the cost, invasiveness and the potential complications prevent many physicians from performing liver biopsies or imaging studies in most patients who are potential candidates for NAFLD. Second, although BMI‐adjusted muscle mass was used for analysis, impaired muscle function precedes decline in muscle mass. As muscle strength and mobility are stronger predictors of long‐term functional decline compared with muscle mass, a study incorporating physical performance, for example, hand grip strength and gait speed, may provide a more comprehensive view of sarcopenia on NAFLD and NAFLD‐related liver cirrhosis in the future.^34^ Moreover, muscle mass was calculated using bioimpedance method, where inherent prediction error exists on an individual level.^19^ Third, participants with concomitant viral liver disease were not excluded from the analysis as serology data were not available.

Notwithstanding these limitations, this study has important strengths. This cohort incorporates a large number of study participants with a median follow‐up of more than 10 years. Also, we have evaluated not only the significance of muscle mass but also the value of genetic polymorphism in assessing incident NAFLD and NAFLD‐related hepatic fibrosis. As genetic predisposition is fundamental to a thorough understanding of NAFLD and its consequences, we believe that this study has the merit of embracing both environmental and genetic factors. Last but not least, we have evaluated not only NAFLD but also hepatic fibrosis in participants with sole NAFLD at baseline. Because NAFLD‐related complications, which exacerbate the burden of NAFLD, increase with the severity of hepatic fibrosis, it is only judicious to determine the risk factors for not only non‐alcoholic fatty liver but also NAFLD‐related hepatic fibrosis.

## Conclusion

Collectively, the findings from this study suggest that maintaining muscle mass and integrating treatments to prevent metabolic syndrome and obesity may be pivotal in preventing NAFLD. In those already diagnosed with NAFLD, it may be beneficial to take into account the *PNPLA3* genetic variant in individualizing surveillance, as it may be more influential in progression to NAFLD‐related hepatic fibrosis than environmental factors.

## Conflict of interest

The authors declare no conflict of interest.

## Funding

This study was supported by Korea National Institute of Health (2022ER090700).

## Supporting information


**Table S1.** Baseline characteristics according to the muscle mass/BMI quartile
**Table S2.** Contribution of genetic risk factors to NAFLD in the fully adjusted model (Model 3)
**Table S3.** Characteristics of participants according to the progression of hepatic fibrosis
**Table S4.** Association of progression to hepatic fibrosis and low muscle mass index (Age < 65)
**Table S5.** Assessment of risk factors to hepatic fibrosis using APRI in the fully adjusted model (Model 3)
**Figure S1.** Cox regression and subgroup analyses of the incident hepatic fibrosis
