# Supplementary material for: Different effects of low muscle mass on the risk of non‐alcoholic fatty liver disease and hepatic fibrosis in a prospective cohort
Source: J Cachexia Sarcopenia Muscle. 2022 Nov 20;14(1):260–9. doi: 10.1002/jcsm.13125 (PMC9891951; doi:10.1002/jcsm.13125)
Supplement: Supplementary file 1 — Table S1. Baseline characteristics according to the muscle mass/BMI quartile Table S2. Contribution of genetic risk factors to NAFLD in the fully adjusted model (Model 3) Table S3. Characteristics of participants according to the progression of hepatic fibrosis Table S4. Association of progression to hepatic fibrosis and low muscle mass index (Age < 65) Table S5. Assessment of risk factors to hepatic fibrosis using APRI in the fully adjusted model (Model 3) Figure S1. Cox regression and subgroup analyses of the incident hepatic fibrosis [file JCSM-14-260-s001.docx]

**Supplementary Table 1. Baseline characteristics according to the muscle mass/BMI quartile**

|  |  |  |  |  |  |  |
| --- | --- | --- | --- | --- | --- | --- |
|  | **Total** | **Q1** | **Q2** | **Q3** | **Q4** | ***P* value** |
|  | N=5,480 | N=1,382 | N=1,365 | N=1,368 | N=1,365 |  |
| Age | 51.5 ± 8.8 | 55.5 ± 8.9 | 52.1 ± 8.6 | 50.5 ± 8.2 | 48.0 ± 7.7 | <0.001 |
| Female (n, %) | 3,087 (56.3) | 772 (55.9) | 773 (56.6) | 773 (56.5) | 769 (56.3) | 0.826 |
| Smoking (pack-year) | **0.0 (10.8)** | **0.0 (10.0)** | **0.0 (13.8)** | **0.0 (10.0)** | **0.0 (11.5)** | **0.240** |
| **Alcohol (g/day)** | **0.0 (3.7)** | **0.0 (2.8)** | **0.0 (3.7)** | **0.0 (4.4)** | **0.0 (4.6)** | **<0.001** |
| **Alcohol duration (year)** |  |  |  |  |  | **0.003** |
| **0 – 5** | **306 (11.8)** | **62 (10.1)** | **78 (12.3)** | **79 (12.0)** | **87 (12.7)** |  |
| **6 – 10** | **283 (10.9)** | **61 (10.0)** | **67 (10.5)** | **79 (12.0)** | **76 (11.1)** |  |
| **11 – 20** | **476 (18.4)** | **100 (16.4)** | **99 (15.6)** | **113 (17.2)** | **164 (23.9)** |  |
| **≥ 21** | **1,525 (58.9)** | **388 (63.5)** | **392 (61.6)** | **385 (58.7)** | **360 (52.4)** |  |
| Hypertension | 1,343 (24.5) | 489 (35.4) | 360 (26.4) | 288 (21.1) | 206 (15.1) | <0.001 |
| Diabetes mellitus | 704 (12.8) | 252 (18.2) | 204 (14.9) | 152 (11.1) | 96 (7.0) | <0.001 |
| Metabolic syndrome | 2,068 (37.7) | 769 (55.6) | 573 (42.0) | 444 (32.5) | 282 (20.7) | <0.001 |
| Obesity (BMI ≥ 25 kg/m^2^) | 2,346 (42.8) | 971 (70.3) | 672 (49.2) | 477 (34.9) | 226 (16.6) | <0.001 |
| Waist circumference (cm) | 81.9 ± 8.8 | 86.4 ± 8.4 | 82.7 ± 8.2 | 80.6 ± 8.0 | 77.8 ± 8.2 | <0.001 |
| BMI (kg/m^2^) | 24.6 ± 3.1 | 26.6 ± 3.1 | 25.0 ± 2.8 | 24.0 ± 2.6 | 22.7 ± 2.4 | <0.001 |
| Muscle mass/BMI | 1.76 ± 0.33 | 1.53 ± 0.26 | 1.70 ± 0.27 | 1.82 ± 0.28 | 2.01 ± 0.30 | <0.001 |
| HbA1c (%) | 5.8 ± 0.9 | 5.9 ± 0.9 | 5.8 ± 1.0 | 5.7 ± 0.9 | 5.6 ± 0.8 | <0.001 |
| Fasting glucose (mg/dL) | 87.1 ± 21.6 | 89.0 ± 21.8 | 88.0 ± 22.5 | 86.3 ± 21.8 | 85.2 ± 20.0 | 0.001 |
| Platelet (×10^3^/μL) | 267 ± 65 | 277 ± 68 | 271 ± 67 | 265 ± 61 | 255 ± 60 | <0.001 |
| Albumin (g/dL) | **4.26 ± 0.33** | **4.24 ± 0.32** | **4.25 ± 0.32** | **4.28 ± 0.34** | **4.27 ± 0.34** | <0.001 |
| Total bilirubin (mg/dL) | **0.62 ± 0.32** | **0.59 ± 0.29** | **0.60 ± 0.30** | **0.63 ± 0.35** | **0.65 ± 0.35** | <0.001 |
| AST (IU/L) | **26 (9)** | **27 (9)** | **26 (8)** | **26 (9)** | **25 (7)** | 0.015 |
| ALT (IU/L) | **22 (14)** | **25 (15)** | **22 (15)** | **22 (13)** | **20 (11)** | <0.001 |
| GGT (IU/L) | **18 (19)** | **22 (26)** | **19 (21)** | **17 (18)** | **15 (14)** | 0.001 |
| Total cholesterol (mg/dL) | 192 ± 36 | 200 ± 37 | 194 ± 36 | 191 ± 37 | 183 ± 33 | <0.001 |
| Triglyceride (mg/dL) | **133 (88)** | **153 (96)** | **141 (95)** | **128 (85)** | **112 (69)** | <0.001 |
| HDL-C (mg/dL) | 44 ± 10 | 43 ± 9 | 44 ± 10 | 44 ± 10 | 46 ± 10 | <0.001 |
| LDL-C (mg/dL) | 116 ± 33 | 121 ± 35 | 117 ± 33 | 116 ± 34 | 111 ± 31 | <0.001 |
| BUN (mg/dL) | 14.3 ± 3.8 | 14.8 ± 4.2 | 14.4 ± 3.8 | 14.2 ± 3.6 | 14.0 ± 3.6 | <0.001 |
| Creatinine (mg/dL) | 0.84 ± 0.20 | 0.84 ± 0.23 | 0.84 ± 0.20 | 0.84 ± 0.18 | 0.85 ± 0.20 | 0.134 |
| TSH (μIU/mL) | 1.70 ± 0.91 | 1.63 ± 0.94 | 1.78 ± 0.86 | 1.75 ± 0.98 | 1.66 ± 0.85 | 0.869 |
| free T4 (ng/dL) | 1.15 ± 0.17 | 1.18 ± 0.18 | 1.13 ± 0.17 | 1.11 ± 0.14 | 1.12 ± 0.18 | 0.043 |
| CRP (mg/dL) | **0.14 (0.18)** | **0.18 (0.21)** | **0.14 (0.19)** | **0.13 (0.18)** | **0.11 (0.14)** | <0.001 |

Data are shown as mean ± standard deviation, median (interquartile range), or number (percentage). BMI, body mass index; HDL-C, high density lipoprotein-cholesterol; LDL-C, low density lipoprotein-cholesterol; BUN, blood urea nitrogen; AST, aspartate aminotransferase; ALT, alanine aminotransferase; GGT, γ-glutamyl transferase; TSH, thyroid stimulating hormone; CRP, C-reactive protein; Q, quartile for muscle mass divided by BMI

**Supplementary Table 2. Contribution of genetic risk factors to NAFLD in the fully adjusted model (Model 3)**

|  |  |  |  |
| --- | --- | --- | --- |
| **Model 3** | **HR** | **95% CI** | ***P* value** |
| Muscle mass/BMI | 1.18 | 1.11 – 1.27 | <0.001 |
| Age | 0.97 | 0.96 – 0.97 | <0.001 |
| Sex | 0.73 | 0.64 – 0.83 | <0.001 |
| Metabolic syndrome | 1.74 | 1.50 – 2.01 | <0.001 |
| Obesity | 3.15 | 2.73 – 3.63 | <0.001 |
| *PNPLA3* (Additive) | 0.99 | 0.90 – 1.09 | 0.881 |
| *TM6SF2* (Dominant) | 1.19 | 1.00 – 1.40 | 0.044 |

HR, hazard ratio; CI, confidence interval; BMI, body mass index

**Supplementary Table 3. Characteristics of participants according to the progression of hepatic fibrosis**

|  |  |  |  |  |  |  |
| --- | --- | --- | --- | --- | --- | --- |
|  | **Baseline** | | | **Follow up** | | |
|  | **Fibrosis (-)** | **Fibrosis (+)** | ***P* value** | **Fibrosis (-)** | **Fibrosis (+)** | ***P* value** |
|  | N=624 | N=552 |  | N=314 | N=443 |  |
| Age | 48.3 ± 7.2 | 52.1 ± 8.0 | <0.001 | 60.6 ± 5.6 | 63.3 ± 7.9 | <0.001 |
| Female (n, %) | 372 (59.6) | 311 (56.3) | 0.256 | 178 (56.7) | 239 (55.2) | 0.685 |
| Smoking (pack-year) | 0.0 (10.0) | 0.0 (10.0) | 0.797 | 0.0 (13.0) | 0.0 (11.3) | 0.589 |
| Alcohol (g/day) | 0.0 (3.5) | 0.0 (3.0) | 0.581 | 0.0 (4.3) | 0.0 (3.5) | 0.913 |
| Alcohol duration |  |  | 0.740 |  |  | 0.586 |
| 0 – 5 | 35 (13.3) | 31 (11.6) |  | 4 (3.1) | 9 (5.0) |  |
| 6 – 10 | 26 (9.8) | 31 (11.6) |  | 5 (3.9) | 10 (5.6) |  |
| 11 – 20 | 57 (21.6) | 51 (19.0) |  | 11 (8.5) | 20 (11.1) |  |
| ≥ 21 | 146 (55.3) | 155 (57.8) |  | 109 (84.5) | 141 (78.3) |  |
| Hypertension | 189 (30.3) | 187 (33.9) | 0.188 | 164 (52.2) | 234 (54.0) | 0.624 |
| Diabetes mellitus | 164 (26.3) | 138 (25.0) | 0.615 | 143 (45.5) | 179 (41.4) | 0.264 |
| Metabolic syndrome | 424 (67.9) | 367 (66.5) | 0.594 | 229 (72.9) | 303 (70.1) | 0.405 |
| Obesity (BMI ≥ 25 kg/m^2^) | 555 (88.9) | 494 (89.5) | 0.761 | 238 (76.3) | 343 (79.6) | 0.282 |
| Waist circumference (cm) | 88.5 ± 7.4 | 89.5 ± 7.2 | 0.016 | 91.4 ± 8.9 | 92.4 ± 8.7 | 0.126 |
| BMI (kg/m^2^) | 27.8 ± 2.5 | 27.9 ± 2.4 | 0.451 | 27.1 ± 3.1 | 27.2 ± 3.0 | 0.337 |
| Muscle mass/BMI | 1.67 ± 0.32 | 1.66 ± 0.32 | 0.567 | 1.63 ± 0.35 | 1.61 ± 0.34 | 0.292 |
| HbA1c (%) | 6.2 ± 1.3 | 6.1 ± 1.0 | 0.070 | 6.5 ± 1.4 | 6.2 ± 1.0 | <0.001 |
| Fasting glucose (mg/dL) | 95.6 ± 32.9 | 92.7 ± 23.2 | 0.017 | 113.6 ± 41.3 | 108.3 ± 26.9 | 0.025 |
| Platelet (×10^3^/μL) | 306.8 ± 63.4 | 273.4 ± 53.9 | <0.001 | 293.1 ± 57.6 | 232.6 ± 47.9 | <0.001 |
| Albumin (g/dL) | 4.29 ± 0.33 | 4.28 ± 0.34 | 0.645 | NA | NA | NA |
| Total bilirubin (mg/dL) | 0.58 ± 0.27 | 0.58 ± 0.28 | 0.838 | NA | NA | NA |
| AST (IU/L) | 26 (10) | 28 (10) | <0.001 | 22 (7) | 26 (8) | <0.001 |
| ALT (IU/L) | 31 (21) | 33 (22) | 0.069 | 25 (13) | 24 (13) | 0.696 |
| GGT (IU/L) | 27 (30) | 27 (27) | 0.730 | NA | NA | NA |
| Total cholesterol (mg/dL) | 205 ± 40 | 203 ± 36 | 0.471 | 186 ± 38 | 180 ± 37 | 0.047 |
| Triglyceride (mg/dL) | 179 (122) | 166 (115) | 0.036 | 138 (83) | 124 (76) | 0.006 |
| HDL-C (mg/dL) | 40 ± 8 | 42 ± 9 | 0.002 | 42 ± 11 | 44 ± 11 | 0.056 |
| LDL-C (mg/dL) | 123 ± 37 | 122 ± 35 | 0.569 | 112 ± 32 | 108 ± 33 | 0.132 |
| BUN (mg/dL) | 14.2 ± 4.6 | 14.9 ± 3.6 | 0.010 | 15.7 ± 4.8 | 16.7 ± 4.6 | 0.005 |
| Creatinine (mg/dL) | 0.85 ± 0.23 | 0.86 ± 0.19 | 0.412 | 0.96 ± 0.21 | 0.99 ± 0.20 | 0.063 |
| TSH (μIU/mL) | 1.45 ± 0.91 | 1.78 ± 1.04 | 0.279 | 2.18 ± 5.41 | 1.97 ± 1.95 | 0.470 |
| free T4 (ng/dL) | 1.17 ± 0.16 | 1.16 ± 0.19 | 0.844 | 1.20 ± 0.18 | 1.17 ± 0.17 | 0.016 |
| CRP (mg/dL) | 0.19 (0.24) | 0.17 (0.20) | 0.053 | 0.87 (1.13) | 0.76 (1.08) | 0.160 |

Data are shown as mean ± standard deviation, median (interquartile range), or number (percentage). NAFLD, non-alcoholic fatty liver disease; BMI, body mass index; HDL-C, high density lipoprotein-cholesterol; LDL-C, low density lipoprotein-cholesterol; BUN, blood urea nitrogen; AST, aspartate aminotransferase; ALT, alanine aminotransferase; GGT, γ-glutamyl transferase; TSH, thyroid stimulating hormone; CRP, C-reactive protein

**Supplementary Table 4. Association of progression to hepatic fibrosis and low muscle mass index (Age < 65)**

|  |  |  |  |  |
| --- | --- | --- | --- | --- |
|  |  | **HR** | **95% CI** | ***P* value** |
| **Unadjusted** | Low muscle mass (Q1) | 1.20 | 1.01 – 1.43 | 0.043 |
| **Model 1** | Low muscle mass (Q1) | 0.99 | 0.83 – 1.19 | 0.950 |
|  | Age | 1.06 | 1.05 – 1.08 | <0.001 |
|  | Sex | 1.30 | 1.09 – 1.56 | 0.004 |
| **Model 2** | Low muscle mass (Q1) | 0.99 | 0.82 – 1.19 | 0.908 |
|  | Age | 1.07 | 1.05 – 1.08 | <0.001 |
|  | Sex | 1.31 | 1.09 – 1.58 | 0.003 |
|  | Metabolic syndrome | 0.83 | 0.68 – 1.00 | 0.055 |
|  | Obesity | 1.06 | 0.78 – 1.43 | 0.712 |
| **Model 3** | Low muscle mass (Q1) | 1.01 | 0.84 – 1.21 | 0.949 |
|  | Age | 1.07 | 1.05 – 1.08 | <0.001 |
|  | Sex | 1.33 | 1.11 – 1.59 | 0.002 |
|  | Metabolic syndrome | 0.83 | 0.69 – 1.00 | 0.050 |
|  | Obesity | 1.06 | 0.79 – 1.43 | 0.707 |
|  | *PNPLA3* | 1.19 | 1.05 – 1.35 | 0.007 |
|  | *TM6SF2* | 1.19 | 0.94 – 1.52 | 0.148 |

HR, hazard ratio; CI, confidence interval

Model 1: adjusted for age, sex

Model 2: adjusted for age, sex, metabolic syndrome, obesity

Model 3: adjusted for age, sex, metabolic syndrome, obesity, *PNPLA3* and *TM6SF2* genotypes

**Supplementary Table 5. Assessment of risk factors to hepatic fibrosis using APRI in the fully adjusted model (Model 3)**

|  |  |  |  |
| --- | --- | --- | --- |
| **Model 3** | **HR** | **95% CI** | ***P* value** |
| Muscle mass/BMI | 1.17 | 0.84 – 1.62 | 0.344 |
| Age | 1.00 | 0.98 – 1.02 | 0.765 |
| Sex | 1.32 | 0.97 – 1.81 | 0.081 |
| Metabolic syndrome | 1.18 | 0.83 – 1.68 | 0.358 |
| Obesity | 0.99 | 0.61 – 1.59 | 0.963 |
| *PNPLA3* (Additive) | 1.13 | 0.90 – 1.42 | 0.303 |
| *TM6SF2* (Dominant) | 1.18 | 0.80 – 1.75 | 0.402 |

APRI, aspartate aminotransferase to platelet ratio index; HR, hazard ratio; CI, confidence interval; BMI, body mass index

Model 3: adjusted for age, sex, metabolic syndrome, obesity, *PNPLA3* and *TM6SF2* genotypes

**Supplementary Figure 1. Cox regression and subgroup analyses of the incident hepatic fibrosis**

**
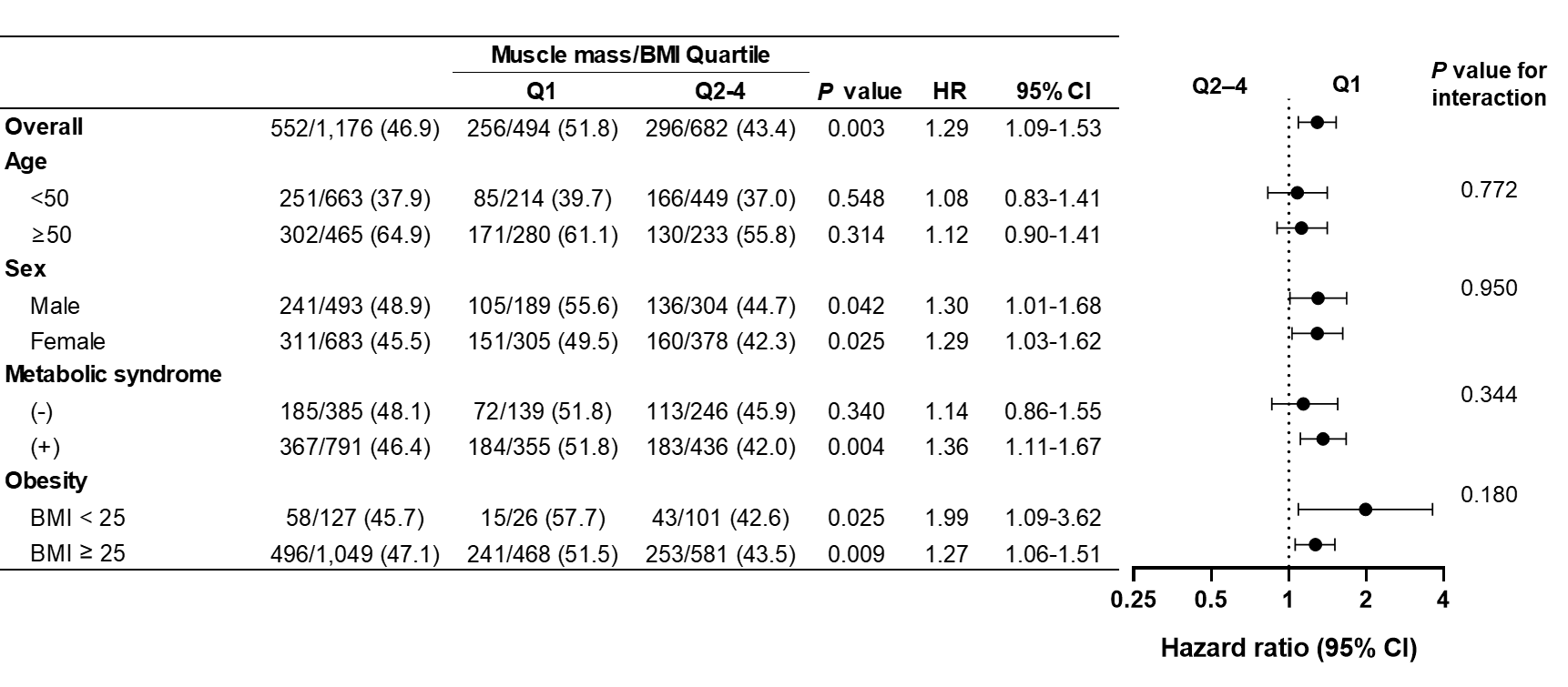
**
